# Application of non-invasive prenatal testing for fetal chromosomal disorders in low-risk pregnancies: a follow-up study in central China

**DOI:** 10.3389/fgene.2025.1574775

**Published:** 2025-06-18

**Authors:** Qiuxiang Huang, Qiao Xu, Meihuan Chen, Wenli Fan, Hailong Huang

**Affiliations:** ^1^ College of Clinical Medicine for Obstetrics and Gynecology and Pediatrics, Fujian Medical University, Fuzhou, China; ^2^ Department of Obstetrics, Wuhan Children’s Hospital (Wuhan Maternal and Child Healthcare Hospital), Tongji Medical College, Huazhong University of Science and Technology, Wuhan, China; ^3^ Fujian Provincial Key laboratory of Prenatal diagnosis and Birth defects, Medical Genetic Diagnosis and Therapy Center of Fujian Maternity and Child Health Hospital, Fuzhou, China

**Keywords:** non-invasive prenatal testing (NIPT), follow-up, low-risk, aneuploidy, copy number variations (CNVs)

## Abstract

**Objective:**

To evaluate the performance and screening value of noninvasive prenatal testing (NIPT) in low-risk pregnancies.

**Methods:**

A retrospective analysis was conducted on 60193 low-risk pregnancies over the last 5 years. Whole-genome sequencing of maternal plasma cell-free DNA was performed using next-generation sequencing. NIPT-positive results were confirmed using amniocentesis with karyotyping and/or copy number variation sequencing and chromosomal microarray analysis. Fetal outcomes were assessed using electronic medical records or telephone calls.

**Results:**

Overall, 598 (0.99%) NIPT-positive cases were identified. The distribution of chromosomal abnormalities included sex chromosome aneuploidies (SCAs; 55.85%), rare autosomal aneuploidies (RAAs; 20.40%), copy number variations (CNVs; 11.20%), trisomy 21 (T21; 6.86%), trisomy 13 (T13; 4.01%), and trisomy 18 (T18; 1.67%). A total of 572 (95.65%) patients with NIPT-positive results underwent amniocentesis, and 55.77% (319/572) cases were confirmed. The positive predictive values (PPV) for T21, T18, T13, SCAs, RAAs, and CNVs were 87.50%, 60.00%, 34.78%, 58.97%, 32.50%, and 69.70%, respectively, and the PPV for the trisomy was higher than that for the X-monomer in SCAs. NIPT-positive results for RAAs were common in T8, T10, T16 and T20, but T16 was the most common true positive result, accounting for 33.33% (13/39) of the cases. The termination rates of true-positive pregnancies were 100% (T21, T18 and T13), 79.49% (RAAs), 67.39% (CNVs) and 78.07% (SCAs).

**Conclusion:**

This study highlights the importance of genome-wide screening based on NIPT in low-risk pregnancies. Prenatal screening by NIPT has a high sensitivity and PPV. Moreover, it can greatly reduce invasive procedures and birth defects.

## 1 Introduction

Birth defects are still recognized as a global public health problem that may cause early miscarriage, stillbirth, neonatal death or defects (Almli et al., 2020). Chromosomal abnormalities are among the most important causes (Feldkamp et al., 2017), including aneuploidy, deletions, duplications, and translocations of varying sizes, with aneuploidy accounting for the majority (American College of Obstetricians and Gynecologists' Committee on Practice Bulletins—Obstetrics Committee on Genetics Society for Maternal-Fetal Medicine, 2020). Since the discovery of cell-free DNA in maternal plasma (Lo et al., 1997), non-invasive prenatal testing (NIPT) has been widely used for prenatal screening because of its high accuracy (van der Meij et al., 2023; Harasim et al., 2022). High sensitivity and specificity of NIPT screening for major fetal aneuploidy abnormalities (trisomy 21, trisomy 18, and trisomy 13) have been demonstrated; however, cell-free fetal DNA is mainly derived from apoptosis of placental trophoblast cells rather than fetal tissues, therefore, placental and maternal abnormalities are potential factors affecting the accuracy of NIPT (Tjoa et al., 2006) and its positive predictive value (PPV) is not very high, especially for other aneuploidies. Rose et al. (2022) reported that PPV of trisomy21 (T21), trisomy18 (T18), and trisomy13 (T13) were 91.8%, 65.8% and 37.2%, respectively, whereas those of the X-monomers in sex chromosome aneuploidies (SCAs) and rare autosomal aneuploidies (RAAs) were lower (Martin et al., 2023; Hu T. et al., 2022). Its relatively low PPV increases the difficulty of prenatal counselling. Whether NIPT can be used for first-tier screening and replace the traditional second-trimester screening remain controversial topics (Ghiasi et al., 2023; Dai et al., 2022; Pandya et al., 2024). Meanwhile, the high cost of NIPT combined with false-positive results and the risk of invasive diagnosis increases anxiety in pregnant women and even causes them to terminate pregnancies (Bakkeren et al., 2024).

At present, large-sample studies on NIPT in low-risk pregnancies are rare, especially for the systematic evaluation of NIPT and analysis of fetal outcomes. Since 2019, NIPT has been adopted as a free program by the Wuhan government, and many low-risk pregnant women have engaged in this program. The performance of NIPT and pregnancy outcomes of low-risk populations in the Wuhan area were retrospectively analyzed with the aim of providing a clinical reference.

## 2 Materials and methods

### 2.1 Study participants

A total of 81389 pregnant women tested by NIPT (except no call results) were included from January 2019 to June 2023 at the Wuhan Maternal and Child Healthcare Hospital. Among them, 60193 women with low-risk fetal abnormalities were recruited as study participants. The maternal age ranged from 18 to 34 years with the average age of 29.82 ± 0.02 years, and the gestational age ranged from 11 to 23 weeks with the average age of 15.38 ± 0.03 weeks. A semi-structured interview was conducted to assess the risk of fetal abnormalities before NIPT, after which the patient information form was filled. Low-risk groups were defined as follows: 1) maternal age <35 years; 2) singleton pregnancy; 3) low risk on maternal serum screening; 4) prenatal ultrasound revealing no abnormalities; 5) no family history of genetic disease; 6) no chromosomal abnormalities observed in the previous pregnancies.

#### 2.1.1 Ethical approval statement

This study was approved by the Ethics Review Committee of Wuhan Maternal and Child Healthcare Hospital (Approval No.2023R019-E01). The study was performed in accordance with the Declaration of Helsinki and informed consent was obtained from all the pregnant women.

### 2.2 Sample collection and processing

#### 2.2.1 Blood sample collecting

All pregnant women underwent NIPT using maternal peripheral blood samples. Approximately 3–5 mL of peripheral blood was collected from the pregnant women. Next, the blood samples were transferred to labelled cell-free DNA (cfDNA) storage tubes (Kangwei Century Biotechnology Co., Jiangsu, China), mixed 8–10 times, and stored temporarily in 4°C refrigerators.

#### 2.2.2 Extraction of cfDNA

Plasma was isolated from blood samples within 72 h of sampling. The blood storage tubes were centrifuged at 1,600 × *g* and 4°C for 10 min. Plasma was collected and dispensed into 2.0 mL Eppendorf tubes and then centrifuged again at 16,000 × *g* and 4°C, for another 10 min. Plasma cfDNA was extracted from the isolated plasma samples using NucleoMag cfDNA isolation kit (BGI-Tech, Wuhan, China) according to the manufacturer’s instructions. After extracting 1.2 mL of plasma, the DNA concentration of 42 uL eluent should be detected within the range of 0.05–0.7 ng/μL. If the concentration is not within the range, the DNA concentration should be extracted again. If the double extraction fails to meet the standard, the blood should be collected again. The cfDNA was amplified with a MiniAmp^™^ PCR kit (Thermo Fisher Scientific, United States).

#### 2.2.3 High-throughput sequencing

A cfDNA library was constructed for sequencing. The cfDNA for sequencing was measured with a Qubit^®^ 3.0 Fluorometer (Thermo Fisher Scientific, United States). Fetal Chromosome Aneuploid (T21, T18, and T13) Detection Kit (BGI-Tech, Wuhan, China) was used for library construction/quality control and library amplification. The resulting libraries were sequenced using the Bioelectronic BGISEQ-2000 sequencing system (BGI-Tech, Wuhan, China) for library construction, quality control, and library amplification, according to the manufacturer’s instructions. The standard *z*-score test for chromosomal aneuploidy in NIPT is defined as

$$Z{\text{score}}_{\text{chr}N}=\frac{\;\%\text{chr}N_{\text{tes}t}-\text{mean}\;\left(\%\text{chr}N_{\text{ref}}\;\right)}{S.D.\left(\%\text{chr}N_{\text{ref}}\right)},\%\text{chr}N_{\text{test}}=\frac{\text{Unique}\;\text{count}\;\text{for}\;\text{chr}N}{\text{Total}\;\text{unique}\;\text{count}},$$

where %chr*N*_test_ represents the proportion of reads for the current chromosome in the test sample relative to all chromosomes. Mean (%chr*N*_ref_) signifies the average proportion of reads for this chromosome in the normal human reference gene group samples relative to all chromosomes, and S.D. (%chr*N*_ref_) denotes the standard deviation of the proportion of reads for this chromosome in the normal human reference gene group samples. Chromosomes with a Z-score between −3 and 3 were defined as being NIPT-negative, otherwise, they were considered NIPT-positive.

### 2.3 Invasive prenatal diagnosis

Amniocentesis (16–24 weeks) is recommended for all pregnant women with positive NIPT results. The collected fetal cells were analyzed by karyotyping and/or copy number variation sequencing (CNV-seq)/chromosomal microarray analysis (CMA) based on clinical indications and the participants’ willingness. If NIPT indicates aneuploidy or copy number variation ≥10 M, karyotyping could be used. However, for more accurate detection, both karyotyping and CNV-seq were recommended. For pregnant women with negative NIPT results, amniocentesis was based on the clinical indications such as fetal ultrasound or clinical abnormalities needed to detect chromosomal disorders. All participants were offered prenatal counselling and provided informed consent.

### 2.4 Pregnancy outcome follow-up

All pregnant women with NIPT-positive results were followed up for 12 weeks *postpartum*. The NIPT-positive pregnant women who refused prenatal diagnosis were followed up until 1 year after delivery. All pregnant women with live births were given a physical examination around 42 days after delivery, along with an examination or telephonic follow-up regarding infant growth and development. Neonates with no abnormalities on postnatal examination were considered negative. NIPT-positive cases with miscarriage, intrauterine fetal death, pregnancy termination before invasive prenatal testing, or in which the pregnancy was continued without invasive testing were excluded from the NIPT performance calculation. For NIPT-negative pregnant women, if there was no obvious abnormality in pregnancy ultrasound, clinical manifestations or prenatal diagnosis, it was considered to be true negative.

### 2.5 Statistical analysis

All data were checked twice and recorded using Microsoft Office Excel format, and the statistical software SPSS 22.0 was used for statistical analysis. The measurement data were expressed as mean ± standard deviation ( $\bar{x}$ ± s), and the difference between the two groups was compared by independent sample t-test. The count data were expressed as the number of cases or composition ratio. *P* < 0.05 was considered statistically significant.

## 3 Result

### 3.1 Detection procedure and NIPT results

**FIGURE 1:**
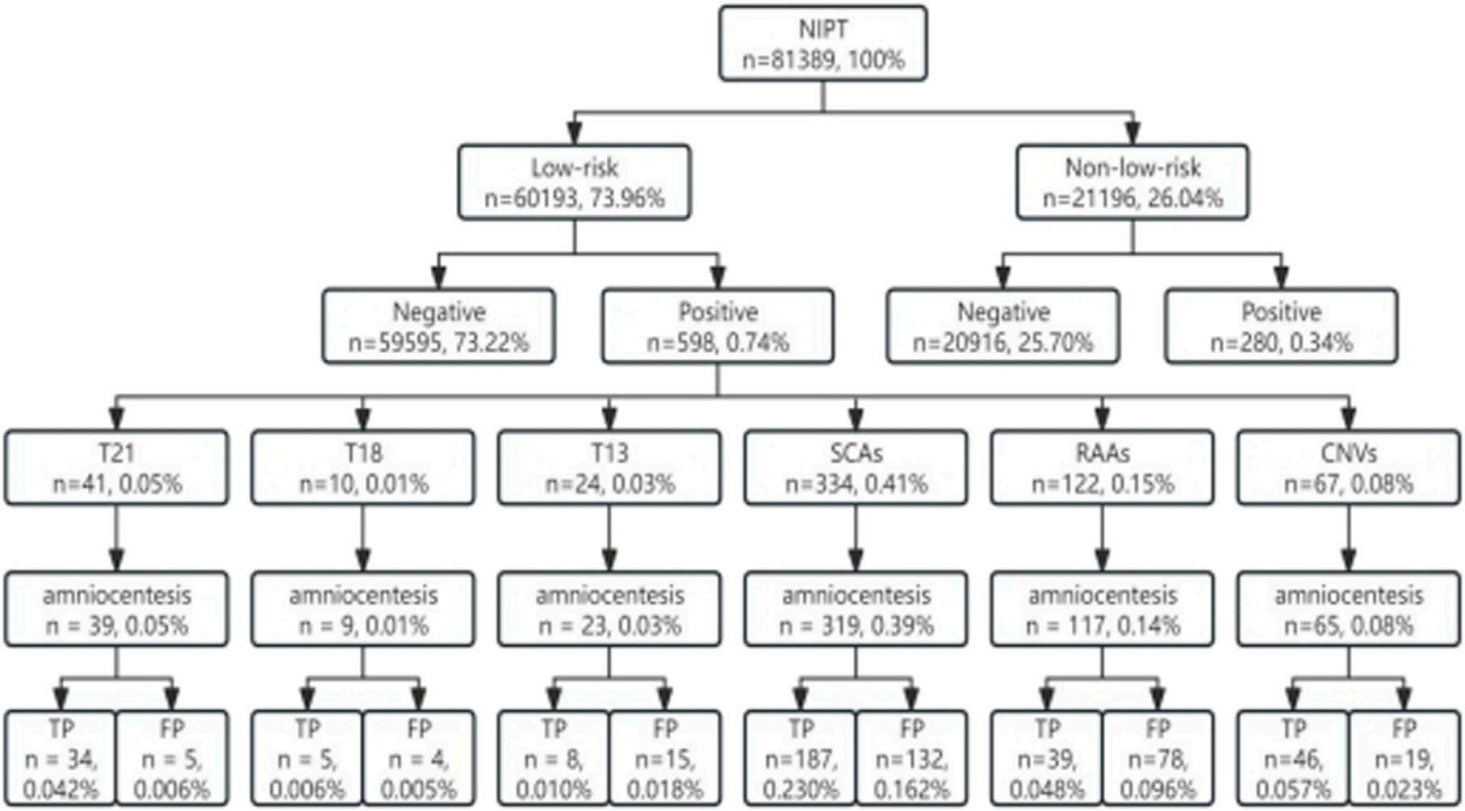
Sample size inclusion flow chart. T21, trisomy 21; T18, trisomy 18; T13, trisomy 13; SCAs, sex chromosome aneuploidies; RAAs, rare autosomal aneuploidies; CNVs, copy number variations; TP, true positive; FP, false positive.

From January 2019 to June 2023, 81389 valid results of pregnant women who underwent NIPT at our hospital (second blood draw rate of 0.67%, 544/81389) were collected, and 60193 is low risk population, 21196 is non-low risk,and the number of low-risk pregnant women was 2.84 times higher than that of non-low-risk pregnant women. Overall, 598 women were tested positive for NIPT in low-risk pregnancies, and 280 pregnancies were tested abnormal in non-low-risk pregnant women (0.99% vs. 1.32%). The distribution of chromosomal abnormalities in NIPT-positive results in low-risk pregnancies was as follows: SCAs (0.41%, 334 cases), RAAs (0.15%, 122 cases), copy number variations (CNVs) (0.08%, 67 cases), T21 (0.05%, 41 cases), T13 (0.03%, 24 cases), and T18 (0.01%, 10 cases). Among them, 95.65% (572/598) were followed up using amniocentesis, and 55.77% (319/572) were confirmed. Overall, 4.35% of the pregnant women with NIPT-positive results (26/598) refused prenatal diagnosis (Figure 1).

### 3.2 The maternal age, gestational age, and fetal fraction distribution of low-risk pregnancies tested by NIPT

**FIGURE 2:**
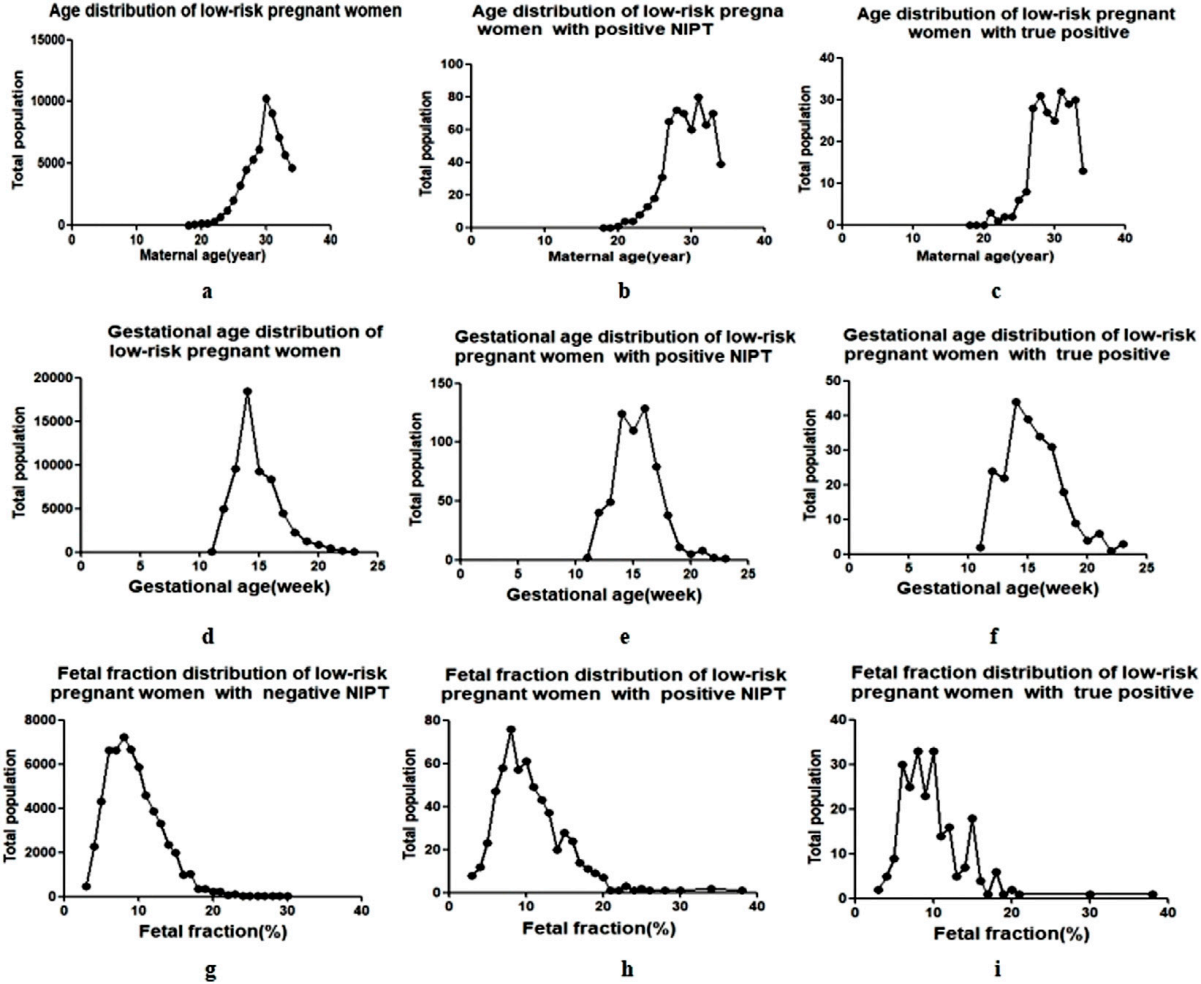
Maternal age and gestational age of NIPT in low-risk pregnant women. The black dots represent the total population of pregnant women (y-axis) plotted against the corresponding maternal age (**(a–c)**, the age interval between each black dot is 1 year), gestational weeks (**(d–f)**, the week interval between each black dot is 1 week) and fetal fraction **(g–i)**, the interval between each black dot is 1%) (x-axis).

The prevalent childbirth age of the low-risk population in Wuhan was 27–33 years, accounting for 79.70% (47960/60193) of the cases, and the normal distribution trend was centered at 30 years of age (Figure 2a). NIPT-positive pregnant women and true-positive cases were also concentrated in this age group (Figures 2b,c), and there was no significant difference between the two groups (Table 1).

**TABLE 1:** Comparison of low-risk pregnancies between NIPT-positive group and true-positive group ( $\bar{x}$ ± s).

Categories	NIPT-positive group	True-positive group	t	P value
Maternal age (year)	29.56 ± 0.12	29.73 ± 0.18	0.78	≥0.05
Gestational age (week)	15.34 ± 0.08	15.44 ± 0.16	0.62	≥0.05
Fetal fraction (%)	11.11 ± 0.24	9.81 ± 0.33	2.82	<0.05

The centralized gestational age during NIPT was 13–16 weeks (Figure 2d), accounting for 75.96% (45723/60193) of the cases, and the prevalent gestational ages of NIPT-positive and true-positive results were 14–17 weeks, accounting for 73.91% (442/598) and 62.70% (200/319) of the cases, respectively (Figures 2e,f). There was also no significant difference in gestational age between NIPT-positive and true-positive groups (Table 1).

The fetal fraction (FF) of male fetuses can be determined by the proportion of Y chromosomes, and the FF of female fetuses can be estimated by building a high-dimensional regression model using the non-uniform distribution of cell-free fetal DNA across the genome, and the QC cutoff for FF was ≥3.5%. The FF in NIPT-negative pregnant women was 3.57%–30.74%, of which 82.34% (49072/59595) were pregnancies with FF of 5%–13% (Figure 2g). The FF in NIPT-positive women and true-positive pregnancies were mainly 5%–16%, accounting for 87.46% (523/598) (Figure 2h) and 91.85% (293/319) (Figure 2i), respectively. The mean FF between the two groups were 11.11 ± 0.24 (%) and 9.81 ± 0.33 (%), showing a statistical difference. (Table 1).

### 3.3 Performance of NIPT for detecting fetal chromosome abnormality in low-risk pregnancies

In addition to the pregnancies of amniocentesis, 16 NIPT-positive pregnant women who had not underwent prenatal diagnosis were tested for fetal tissue after delivery, of which 9 cases were true positive (1 case T21,1 case T18, 2 cases XXX and 5 cases XXY) and 7 cases were false positive (1 case XXX, 2 cases XXY, 3 cases RAAs, 1 case CNVs). Since most NIPT-negative pregnant women were normal, if there were no obvious clinical indications, it was considered to be true negative (including lost to follow-up pregnant women). The results were included in the statistics of NIPT detection efficacy.

The sensitivity and specificity of NIPT in detecting fetal aneuploidy in low-risk pregnancies were 100% and >99%, respectively, whereas those in detecting CNVs were 95.83% and 99.97%, respectively. The total PPV of NIPT was 55.78%. PPVs for T21, T18, T13, SCAs, RAAs, and CNVs were 87.50%, 60.00%, 34.78%, 58.97%, 32.50%, and 69.70%, respectively. The PPV for trisomy in SCAs was higher than that of the X-monomer at 64.81% (XXX), 86.30% (XXY), 82.69% (XYY), and 35.33% (XO). The total false positive value (FPV) of NIPT was 0.43%, and the FPVs for SCAs and RAAs were 0.22% and 0.13%, respectively, which were significantly higher than those for common autosomal trisomies (T21, 0.01%; T18, 0.01%; T13, 0.02%) and CNVs (0.03%). Two false negative patients were found: a heterozygosity deletion of chr16 with a fragment size of 688.80 kb (pathogenic) and a small fragment deletion of chr16 with a fragment size of 1.7 Mb (suspected pathogenic). Both cases were confirmed by amniotic fluid extraction during rivanol amniotic cavity injection after intrauterine fetal death in late pregnancy (30 weeks and 29 weeks of gestation) (Table 2).

**TABLE 2:** Performance of NIPT for detecting fetal chromosome abnormality in low-risk pregnancies.

Chromosome abnormality	Positive n	TP n	FP n	TN n	FN n	Sensitivity % (95%CI)	Specificity % (95%CI)	PPV % (95%CI)	NPV % (95%CI)	FPR %	FNR %
T21	41	35	5	60157	0	100.00 (0.88–1)	99.99 (0.99–0.99)	87.50 (0.72–0.95)	100.00 (0.99–1)	0.01	0.00
T18	10	6	4	60187	0	100.00 (0.52–1)	99.99 (0.99–0.99)	60.00 (0.27–0.86)	100.00 (0.99–1)	0.01	0.00
T13	24	8	15	60184	0	100.00 (0.60–1)	99.98 (0.99–0.99)	34.78 (0.17–0.57)	100.00 (0.99–1)	0.02	0.00
SCAs	334	194	135	59994	0	100.00 (0.98–1)	99.78 (0.99–0.99)	58.97 (0.53–0.64)	100.00 (0.99–1)	0.22	0.00
XO	150	53	97	60140	0	100.00 (0.92–1)	99.84 (0.99–0.99)	35.33 (0.28–0.44)	100.00 (0.99–1)	0.16	0.00
XXX	55	35	19	60157	0	100.00 (0.88–1)	99.97 (0.99–0.99)	64.81 (0.51–0.77)	100.00 (0.99–1)	0.03	0.00
XXY	77	63	10	60126	0	100.00 (0.93–1)	99.98 (0.99–0.99)	86.30 (0.76–0.93)	100.00 (0.99–1)	0.02	0.00
XYY	52	43	9	60150	0	100.00 (0.90–1)	99.99 (0.99–0.99)	82.69 (0.69–0.91)	100.00 (0.99–1)	0.01	0.00
RAAs	122	39	81	60152	0	100.00 (0.89–1)	99.87 (0.99–0.99)	32.50 (0.24–0.42)	100.00 (0.99–1)	0.13	0.00
CNVs	67	46	20	60144	2	95.83 (0.85–0.99)	99.97 (0.99–0.99)	69.70 (0.57–0.80)	100.00 (0.99–0.99)	0.03	9.09
dup	39	26	13	60167	0	100.00 (0.84–0.99)	99.98 (0.99–0.99)	66.67 (0.50–0.80)	100.00 (0.99–1)	0.02	0.00
del	28	20	7	60170	2	91.91 (0.69–0.98)	99.99 (0.99–0.99)	74.07 (0.53–0.88)	100.00 (0.99–0.99)	0.01	22.22
Total	598	328	260	59853	2	99.39 (0.97–0.99)	99.57 (0.99–0.99)	55.78 (0.52–0.60)	100.00 (0.99–0.99)	0.43	0.76

TP, true positive; FP, false positive; TN, total negative; FN, false negative; PPV, positive predictive value; NPV, negative predictive value; FPR, false positive rate; FNR, false negative rate; CI, confidence interval; T21, trisomy 21; T18, trisomy 18; T13, trisomy 13; SCAs, sex chromosome aneuploidies; RAAs, rare autosomal aneuploidies; CNVs, copy number variations; dup, duplication; del, deletion.

### 3.4 Detection and distribution of RAAs in low-risk pregnancies

**FIGURE 3:**
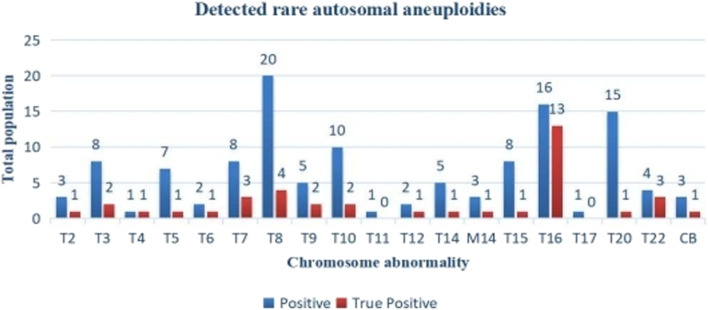
Distribution of NIPT positive and true positive of RAAs. T, trisomy; M, monosomy; CB; Complex abnormality. There were no rare autosomal aneuploidies observed on chromosomes 1 and 19.

Among the 122 cases of RAAs with NIPT positive results, all chromosomes were involved except chr1 and chr19. T8, T10, T16 and T20 were the most common, accounting for 50.00% (61/122) of the cases. Three cases were found to have two combined chromosomal abnormalities (T3 and T16, T8 and T14, and T8 and T20), and three were monosomic (all were chr14). The prenatal diagnosis rate of RAAs was 95.90% (117/122), and the detection rate varied greatly because of the difference in the diagnosis rates among different chromosomes. The true positive RAAs cases were almost mosaic fetal trisomy of different degree, not total trisomy (regardless of the size of the mosaic ratio, as long as the abnormal chromosome indicated by prenatal diagnosis was the same as NIPT, it was considered to be true positive); In the true positive pregnancies, T16 was the most common, accounting for 33.33% (13/39), and one combined T8 and T14 (Figure 3).

### 3.5 Follow-up of fetal outcomes in low-risk pregnancies with NIPT-positive results

All NIPT-positive pregnant women were followed up for pregnancy outcomes, but 9.85% (5871/59595) of NIPT-negative pregnancies were lost to follow-up. The termination rates of true-positive pregnancies were 100% (T21, T18 and T13), 79.49% (RAAs), 67.39% (CNVs) and 78.07% (SCAs). Among the SCAs, 41 pregnancies with true-positive results chose to deliver. Among women with true-positive RAAs, 79.49% (31/39) terminated pregnancies, three delivered after they were diagnosed with uniparental disomy, five delivered with unclear clinical significance. Among the CNVs true positive pregnant women, 31 cases were pathogenic or suspected pathogenic (termination of pregnancy), 13 cases were of unknown clinical significance (12 delivery, one termination of pregnancy after premature rupture of membrane at 20 weeks of gestation), and two cases were of maternal origin (normal maternal phenotype, all delivery). Among all false positive pregnancies, one was intrauterine fetal death, one was terminated owing to family factors, and the rest were delivered, with a delivery rate of 98.90% (179/181). Among 26 NIPT-positive pregnant women without prenatal diagnosis, 16 pregnancies were tested after birth or termination of pregnancy, of which 2 pregnancies were terminated (T21 and T18, both true positivity); and there were 14 live births, 10 SCAs (7 true positive and 3 false positive), 3 RAAs (all negative), and 1 CNVs (clinical significance unknown). Maternal blood tests were normal in 2 cases (T21 and T18). The other 10 cases were not tested due to cost or other reasons and all pregnancies were terminated except for 3 deliveries (2 case SCAs and 1 case RAAs). No significant abnormality was found in all neonates during our follow-up (Table 3).

**TABLE 3:** Fetal outcomes of low-risk pregnancies with NIPT-positive result.

Chromosome abnormality	Amniocentesis results	n	Termination of pregnancy	Stillbirth	Childbirth
T21, T18, T13	TP	47	43	4	0
	FP	24	1	0	23
	Refuse	4	3	1	0
SCAs	TP	187	146	0	41
	FP	132	1	0	131
	Refuse	15	3	0	12
RAAs	TP	39	31	0	8
	FP	78	0	1	77
	Refuse	5	1	0	4
CNVs	TP	46	31	0	15
	FP	19	0	0	19
	Refuse	2	1	0	1
Total		598	261	6	331

T21, trisomy 21; T18, trisomy 18; T13, trisomy 13; SCAs, sex chromosome aneuploidies; RAAs, rare autosomal aneuploidies; CNVs, copy number variations.

## 4 Discussion

Although the risk of fetal aneuploidy increases with maternal age (Yamada et al., 2018; Huang et al., 2024), most pregnant women are under 35. Therefore, birth defects also account for a certain proportion of children born with low-risk pregnancies. This phenomenon was confirmed in the present study. The number of low-risk pregnant women in our study was 2.84 times higher than that of non-low-risk pregnant women. A lower rate of fetal abnormalities was detected by NIPT in low-risk pregnancies than in non-low-risk pregnancies (0.99% vs. 1.32%); however, the total population with NIPT-positive results in low-risk pregnancies were twice as high as that in non-low-risk pregnancies. This study showed that the majority of the pregnant women in Wuhan were 27–30 years old, with both NIPT-positive results and true-positive cases predominantly occurring in this age group. Therefore, the number of fetal abnormalities was closely related to the number of pregnant women tested.

Hence, identifying a suitable prenatal screening method for low-risk populations is particularly important. Despite concerns about NIPT as a first-tier screening tool (Bunnik, 2022; Rehmann-Sutter et al., 2023), some countries, such as the Netherlands, have begun its implementation (Bilardo, 2021), and some studies have recommended NIPT as a publicly funded project (Bunnik et al., 2020). Because of the government funding, NIPT has also been adopted as the first choice of prenatal screening for all pregnant women in Wuhan. Cell-free fetal DNA can be detected in the maternal peripheral blood as early as 4–5 weeks of gestation, and NIPT can be performed at 9 weeks of gestation, although fetal fraction at 10 weeks and beyond are associated with lower test failure rates (Chan et al., 2004). In our study, although the fetal fractions ranged from 3.57% to 38.06%, the fetal scores were concentrated between 5% and 16%, regardless of NIPT-negative, NIPT-positive or true positive results. However, the fetal fractions of NIPT-positive group and true-positive group were statistically different and whether it indicated that true positive results can be distinguished needs further study. The secondary blood drawing rate of NIPT was 0.67% and the detection weeks were concentrated at 13–16 weeks, with the positivity rate concentrated at 14–17 weeks. Indeed, NIPT is not equivalent to diagnostic testing, and the detection time of NIPT should be considered along with the timing of prenatal diagnosis.

Despite the presence of no-call results and secondary blood draws (Guy et al., 2021; Suzumori et al., 2019), cfDNA is still considered the most sensitive and specific screening method for fetal chromosome aneuploidy (Allyse and Wick, 2018). The ACMG strongly recommend NIPT as a highly accurate screening method for T21, T18, and T13 as well as for fetal sex chromosome aneuploid in both singleton and twin pregnancies (Dungan et al., 2023). In our study, the sensitivity and specificity of NIPT were >99%, and the PPVs for T21, T18, and T13 were 87.50%, 60.00% and 34.78%, respectively, which were much higher than those observed in traditional serological screening (Wald et al., 1999).

In this study, most pregnancies with NIPT-positive results had SCAs, followed by RAAs and CNVs. Therefore, screening for chromosomal abnormalities other than T21, T18, and T13 is equally important. The PPV for the trisomy in SCAs was significantly higher than that for the X-monomer, which is consistent with the findings of Kim et al. (2024). The PPV of RAAs was 32.50%, slightly higher than that found in other reports (Pedrola Vidal et al., 2024; Mossfield et al., 2022), which may be related to the statistical criteria. In this study, as long as the abnormal chromosome type of RAAs detected by prenatal diagnosis was the same as that detected by NIPT, it was considered to be true positive for NIPT (regardless of the proportion of mosaic, rather than only total trisomy). This may overestimate the PPV of RAAs. Due to the differences in chromosome mosaic fragments and proportions, fetal phenotypes are also different, and it is difficult to determine a uniform standard. Therefore, true positive of RAAs in this study did not equate to fetal pathogenicity. Whether the fetus was pathogenic required further genetic counseling. In addition, the small sample size of RAAs in this study also affected the performance of NIPT.

The positive results of RAAs were mainly mosaic trisomies, among which T8, T10, T16, and T20 were the most common. Monomers accounted for only 2.46% (3/122), suggesting a higher incidence of trisomies. T16 was the most common among the true-positive cases, and the two false negative cases were found with a chr16 deletion, which may indicate that chr16 abnormality is more common in RAAs in Wuhan. Spinillo et al. (2022) also proposed the importance of chr16. The PPV of NIPT for CNVs was 69.70% in our study, which was slightly higher than that reported in other studies (Wang et al., 2021; Hu Y. et al., 2022), which may be related to the large abnormal fragments detected during the screening. In this study, 80.59% (54/67) of abnormal fragments >5 Mb were detected, and the PPV for the CNVs was closely related to the size of the detected fragments (Chen et al., 2021; Xue et al., 2022). Li et al. (2024) also reported that the PPV for CNVs (>10 Mb) was 66.67%. In general, false positive results of NIPT are common, especially for non-target chromosomal diseases, which might be associated with maternal mosaism or restricted placental mosaism (Acreman et al., 2023; Wang et al., 2014), and further genetic counselings are needed. Meanwhile, maternal blood and placental chromosome testing are recommended for NIPT-positive pregnant women.

In our study, the performance of NIPT in low-risk pregnancies was analyzed comprehensively and systematically using a large sample, and fetal outcomes were followed up, which have not been reported to date. Our study showed that fetal outcomes were closely related to the type of chromosome involved. T21, T18, and T13 have a great impact on fetal quality of life, and 51 participants with true-positive results or those who refused amniotic diagnosis chose to terminate the pregnancies. SCAs have little effect on fetal survival and usually do not occur until puberty (Deng et al., 2021). A total of 53 participants with SCAs with true-positive results or without a prenatal diagnosis chose delivery in our study, and no significant abnormalities were found at birth or during *postpartum* follow-up.

Among the patients with true-positive RAAs, eight chose to give birth and they underwent whole-exon sequencing again, indicating uniparental diploidy or no clear clinical significance; Four participants chose to give birth after refusing amniocentesis; two of whom were diagnosed with fetal growth restriction (T6 and T8), and the others (T5 and T16) underwent *in vitro* fertilization, with no obvious abnormalities after birth. The CNVs phenotype is complex and related to the types and fragments of the chromosomes involved (Parchem et al., 2018; Cai et al., 2022; Liu et al., 2023). In this study, 32.61% (15/46) of true-positive cases of CNVs were delivered; among these, two cases had deletion of chromosome X and origin mother; one of the other two cases had chr8 duplication, and one had chr3 duplication with all involved segments <10 Mb. No abnormalities were found after birth. Although false-positive NIPT results have recently been found to be associated with adverse pregnancy outcomes (Eggenhuizen et al., 2021), all false-positive pregnant women had live births in our follow-up, except for one stillbirth and one termination of pregnancy due to family factors. In addition, the higher rate of pregnancy termination was found with chromosomal abnormalities even for SCAs and CNVs with uncertain phenotypic consequences other than T21, T18, and T13, and Bunnik et al. (2020) reported that the expansion of NIPT had raised ethical issues. Unfortunately, eight participants with NIPT-positive pregnancies without amniocentesis chose to terminate the pregnancy directly, a phenomenon that has also been reported previously (Perrot and Horn, 2022). Therefore, providing timely psychological and genetic counselling is important.

Although our study is the first systematic large-sample study to evaluate the performance of NIPT in low-risk pregnancies in Wuhan, it still has some limitations. Firstly, the follow-up rate in our study was poor; 9.85% of the women with NIPT-negetive results were lost to follow up, which might have affected the evaluation of NIPT performance. This might be because most NIPT-negative pregnant women were normal and lacked sufficient attention. However, according to the previous studies, it is not uncommon for 20%-30%of pregnant Chinese women to be lost to follow up (Xu et al., 2020; Lu et al., 2020). It may be related to the fact that our hospital is a designated hospital for free NIPT, and some women only accepted NIPT but not antenatal care in our hospital. Secondly, the incidence of fetal chromosome abnormalities in each category was small, which may be related to morbidity. Therefore, large-scale multi-center studies are required. Thirdly, due to different chromosomal abnormalities, the onset time and phenotypic severity are different, and our short follow-up time might result in some false negative results, especially for abnormal results other than the target disease, and regular follow-up after birth is also necessary.

In conclusion, our study further demonstrated the high sensitivity and specificity of NIPT, and whole genome screening based on NIPT in low-risk pregnant women is warranted. Birth defects and the risks of invasive diagnosis are significantly reduced by the high PPV of NIPT. Meanwhile, our tracking of pregnancy outcomes also provided a basis for prenatal counseling; however, the number of positive cases for some chromosomal disorders was insufficient, and some patients were lost to follow-up; therefore, further multi-center and large-sample studies are needed.

## Data Availability

The datasets generated and/or analysed during the current study are available at NCBI under project ID J-DS001296-001.
